# Rheological Investigation of Hydroxypropyl Cellulose–Based Filaments for Material Extrusion 3D Printing

**DOI:** 10.3390/polym14061108

**Published:** 2022-03-10

**Authors:** Yee Mon Than, Sarisa Suriyarak, Varin Titapiwatanakun

**Affiliations:** 1 Department of Pharmaceutics and Industrial Pharmacy, Faculty of Pharmaceutical Sciences, Chulalongkorn University, Bangkok 10330, Thailand; yeemonthan23@gmail.com; 2 Department of Food Technology, Faculty of Science, Chulalongkorn University, Bangkok 10330, Thailand; 3 Emerging Processes for Food Functionality Design Research Unit, Chulalongkorn University, Bangkok 10330, Thailand

**Keywords:** viscosity, shear thinning, viscoelastic properties, material extrusion 3D printing, hydroxypropyl cellulose

## Abstract

The rheological properties of drug–polymer mixtures have a significant influence on their processability when using transformative techniques, such as hot-melt-extrusion and material-extrusion 3D printing; however, there has been limited data on printable systems. This study investigated the rheological properties of 17 formulations of successful printed tablets for both immediate and controlled release. Hydroxypropyl cellulose was used in various ratios to obtain printable filaments in combination with various drugs (indomethacin or theophylline), polymers and disintegrants. The complex viscosity, shear thinning behavior and viscoelastic properties were affected by the drug load, polymer composite, disintegrant type, temperature and shear rate applied. Larger windows of processing viscosity were revealed. The viscosity of the printable blends could be as low as the range 10–1000 Pa·s at 100 rad/s angular frequency. All formulations showed shear thinning behavior with a broad slope of complex viscosity from −0.28 to −0.74. The addition of 30–60% drug or disintegrant tended to have greater viscosity values. While microcrystalline cellulose was found to be an alternative additive to lower the storage and loss modulus among disintegrants. This rheological data could be useful for the preformulation and further development of material-extrusion 3D-printing medicines.

## 1. Introduction

Material-extrusion three-dimensional (3D) printing (also known as Fused Filament Fabrication, FFF; or Fused Deposition Modeling, FDM), a part of additive manufacturing (AM), has been extensively applied to fabricate a wide range of drug-delivery devices via layer-by-layer deposition of fused feedstock filament [1,2] on top of spare parts manufacturing (e.g., aerospace, automotive components [3], metal alloys [4] or maritime industries [5,6]). AM cannot only execute production by using various optimization approaches in designs but also can reduce the production cost of complex components [7]. Nowadays, polymers have several commercial applications due to their intrinsic properties, including strength, easy availability, cost, and thermal and chemical resistance [8]. In the pharmaceutical field, a number of polymer mixtures, pharmaceutical excipients (insoluble filler, plasticizer, antioxidant, lubricant, disintegrant and natural fiber [9]) and drug content were melt-extruded to modify the performance of polymer composites, leading to improved printability and the desired drug-release profile [10,11].

Hot melt extrusion (HME) offers intensive mixing and a high temperature to produce different solid compositions, ranging from molecularly dispersed solid solution to a solid drug–polymer suspension [12], and to improve the drug’s bioavailability through increasing the dissolution rate [13,14,15,16]. The successful extrusion of solid dosage forms depends on the flow behavior of the drug substances and the nature of the dispersed systems. Preferably, the dosage form size is kept at a suitable level; thus, the drug-to-excipient ratio should be optimized for orally administered high-dose drugs. Melt processing of such highly viscous dispersions or highly concentrated drugs faces many challenges. For example, interactions between dense solid particles in concentrated suspensions can cause the formation of a reversible structural network, which requires more energy to be crushed down before any deformation can happen upon the applied stress [17]. Meanwhile partly miscible, insoluble or supersaturated drug–polymer mixtures may display apparent yield stress, whereby their viscosity at low shear rates is very high, approaching solid-like behavior [18]. On the other hand, the small molecules of drugs, dissolved into the molten polymers, plasticize the mixture and decrease the viscosity [19]. Thus, polymeric components with different drug loads and drug solubilities tend to have characteristic viscosity, degrees of shear thinning and thermal stability [18].

Recently, an extrusion printer compliant with good manufacturing practices was developed for research and clinical manufacture. Animal studies of printed dosage forms were investigated [20]. However, hitherto, one challenge is to prepare printable filaments by HME as certain force is applied to the filament while being pulled down by the feeding gears toward the heated nozzle during printing [21,22,23]. The melting behavior of composite filament and process parameters have a significant impact on successful material extrusion printing. Printable filament production is performed based on a trial-and-error approach, which is time-consuming and resource-intensive. The non-printable filaments are destroyed. This could be a key barrier when developing expensive materials, including drugs and polymers. Therefore, an efficient approach is needed to screen the printable formulation and assess the feasibility of polymer mixtures for the printing purpose of specific delivery systems, dose adjustment, drug-release characteristics, improved medicine access and personalized medicines. Thermal analysis was used to understand the polymer composites, but the mechanical factor was neglected. A texture analyzer was applied, and the breaking stress and breaking distance of the successful printed filaments were studied to determine the optimal extrusion range [24,25,26]; however, the mechanical measurement still does not mimic the thermal factor in the extrusion process [18].

Rheology is versatile and can be used for material characterizations, including polymer crystallization, mechanical properties, stability and degradation [27,28,29]. Meanwhile, it can be a screening tool to predict the success of composite polymers in the printing process [30], as it provides important information for material extrusion, and a rheometer can mimic the mode of action in terms of heat and shearing in the extrusion process [31,32]. Recent studies have demonstrated the correlation between rheological data and the performance of the materials used during the 3D-printing production [33,34]. Moreover, some research has applied rheological data of feedstock filaments and compared them to the properties of the final product [35,36] because the rheology can influence the material macrostructure [37,38]. An attempt to attach in-line rheological sensors to the printing nozzle was made for real-time monitoring [39]. Nevertheless, the rheological properties of filaments toward printability remain unclear and underutilized.

The rheological properties of the polymer melt play an essential role in determining the optimal conditions of the extrusion and the properties of the extruded object. The extrudability/printability of filaments and the reproducibility of the extruded/printed structures depend on the polymer’s response to extrusion, the polymer’s ability to adhere to previously printed layers and the firmness of the weight of the subsequent layers [10], which were controlled by the material flow [40], temperature of the melt through viscous dissipation [40,41] and shear rate [40,42]. Previous research has studied the relationship of temperature and the structural morphology [43,44], and the effect of bonding formation coupled with microstructure observation, using multiscale damage analysis [45]. Notably, material-extrusion printing is an extension of HME, but the mode of shearing differs between these two technologies, due to the nozzle size and processing speed. Moreover, the shear-thinning behavior of the mixtures can control their ability to be pushed through a nozzle at a specified temperature and the ability to restructure the objects after extrusion [15]. Hence, mixtures should possess characteristic viscosity, shear-thinning behavior and viscoelastic properties on solidifying–liquefying and adhesion to a substrate, which could be useful to predetermine the suitability of filament and assess the feasibility of polymers for material extrusion printing.

There has been limited research on the rheological properties of polymeric systems for material extrusion 3D printing, for example, polyvinylpyrrolidone-vinyl acetate [46], polymethacrylate [47,48], polycaprolactone [49], polylactic acid [50] and polyethylene oxide [32], while polymer mixtures with combined polymers and quaternary blends have been underexplored. Moreover, polymer composites containing hydroxypropyl cellulose have a high potential to fabricate 3D-printed medicines, as there have been a number of published articles [24,51,52,53,54,55,56,57,58,59]. Therefore, the present study aimed to investigate the rheological properties in terms of viscosity, shear-thinning behavior and viscoelastic properties of 17 hydroxypropyl cellulose-based mixtures, which were successfully printed in the form of immediate and controlled release delivery systems [25,26]. Additional polymers (Soluplus^®^, Kollidon^®^ VA 64 or Eudragit^®^ EPO, RS or RL) and/or disintegrants (sodium starch glycolate, microcrystalline cellulose, croscarmellose sodium, crospovidone or low substituted hydroxypropyl cellulose) were combined into the feedstock filament at various ratios, as presented in Table 1. Two model drugs (indomethacin and theophylline) were used.

## 2. Materials and Methods

### 2.1. Materials

Hydroxypropyl cellulose-L (MW 140,000 g/mol; HPC) from Nippon Soda Co., LTD. (Tokyo, Japan) was used. Eudragit^®^ RS PO and Eudragit^®^ RL PO (MW 45,000 g/mol, Röhm Pharma, GmbH, Darmstadt, Germany; Eu RS and Eu RL), semi-crystalline Kollidon^®^VA64 (MW 67,000 g/mol; PVP/VA) and Soluplus^®^ (MW 120,000 g/mol; SLP) were purchased from BASF FE (Ludwigshafen, Germany). Sodium starch glycolate (SSG), croscarmellose sodium (CCM), crospovidone (CrosPVP), microcrystalline cellulose (Avicel PH-101, FMC Biopolymer Co., Ltd., Philadelphia, PA, USA; MCC) and low substituted hydroxypropyl cellulose (LH-31, hydroxypropoxy content = 11%; Shin-Etsu Co., Ltd., Niigata, Japan; L-HPC) were used as disintegrants in this study. Theophylline (THY) and indomethacin (IMC) were obtained from Sigma-Aldrich (St. Louis, MO, USA).

### 2.2. Preparation of Printable Filaments for Material Extrusion Printing

Filaments were prepared according to a protocol performed in our laboratory and the methodology described by Than [25,57]. Pre-mixed physical mixtures (20 g) were prepared using a mortar and pestle for 15 min and fed with a gravimetric feeder. Extruded filaments were fabricated using a single screw extruder (Noztek^®^, Shoreham-by-Sea, England, 1.75 mm diameter nozzle) with specific rotating screw speed (35–45 rpm) and extrusion temperature (135–160 °C) adjusted to the formulation compositions to control the filament diameter. In this work, HPC was used as the main polymer to tailor the immediate and controlled release profiles. Different ratios of polymer–polymer and polymer–disintegrant, including type of drug and drug loading, were investigated for printability and desired properties of the finished products. The optimized compositions of the printable formulations and extrusion temperatures and screw speed are illustrated in Table 1. Filaments were kept in sealed plastic bags and stored in a desiccator at room temperature until further characterizations. The 3D-printed tablets were successfully produced by using a commercial-material-extrusion 3D printer, MakerBot Replicator 2× (MakerBot Inc., Brooklyn, NY 11201, USA), with a dual nozzle of 0.4 mm diameter [25,26]. The printing temperature was set at 200 °C for all formulations.

### 2.3. Rheological Measurement

The viscosity, shear thinning behavior and viscoelastic properties of the printable filaments, as a function of temperature and frequency, were studied by using a HAAKE MARS III rotational rheometer (Thermo Fisher Scientific, Schwerte, Germany), equipped with a 25 mm parallel plate. Samples (500 mg) were weighed and compressed into a disc (25 mm in diameter and 1 mm thickness). The slug was placed in between two flat plates of 25 mm after calibration of the 0.9 mm gap. An oscillatory strain sweep was conducted to determine the linear viscoelastic region (LVR) at a high printing temperature of 200 °C by gradually increasing the amplitude strain from 0.01 to 100%, with a fixed frequency of 1 Hz, since the linear viscoelastic region is generally narrower at a low temperature [27]. The working amplitude strain of 0.5% (within the LVR) was used for all polymeric blends. Then, the oscillatory temperature sweep tests were conducted in the temperature range from 150 to 200 °C, with increments of 10 °C. Other testing conditions used for the oscillation temperature sweep were a strain of 0.5% and fixed angular frequency of 1 Hz. In the oscillation frequency sweep experiment, the angular frequency ranged from 0.02 to 15.924 Hz at fixed temperatures of 150, 160 and 200 °C, and the oscillation strain was kept at 0.5%. The samples were equilibrated for 120 s at each temperature of the experiment. Complex viscosity, storage modulus (G’), loss modulus (G”) and the crossover point of the samples were analyzed.

## 3. Results and Discussion

Rheology is a technique to measure the flow and deformation behaviors of polymeric melts at various temperatures and rates of shear, where viscosity is the measurement of resistance to flow [27]. In other words, temperature and shear rate factors control melting, flow and deformation behavior of materials during melt extrusion [60,61]. In HME, materials are passed through the rotating screws at a high temperature, where they are subjected to a high shear rate, whereas, in material-extrusion printing, materials are fed through the tiny hot tip with the shear of the piston-like push from the driving gear. Small amplitude oscillatory rheological experiments were conducted to evaluate the temperature and frequency (oscillatory shear) dependency of the polymeric blends that could explain the HME-and-material-extrusion printing melt behavior [51].

### 3.1. Oscillatory Shear Analysis

The small amplitude oscillatory shear (SAOS) measurements were carried out at the specified temperature (200 °C) applied for the material extrusion. The periodic concerned flow field in the SAOS differentiates the material viscoelastic response into two components: the elastic in-phase (G’) and the viscous out-of-phase (G”) responses [62]. SAOS is, therefore, of much greater relevance in the characterization of polymers and determines the threshold of linear viscoelastic region (LVER) as a preliminary study to explore an appropriate range of strain values for the samples and to ensure that the analysis was well-suited with the theoretical constraints. The amplitude sweep, where the shear storage and loss moduli were recorded as a function of the shear strain to define the limits of the applicability of the linear viscoelastic theory [63] at a fixed angular frequency, was conducted. In this LVER, molecules have sufficient time to relax through Brownian motion and the polymer structure remains unchanged as the entangled and coiled state when a very small deformation is applied to the polymer melt, or when the deformation rate is very slow [27].

**Figure 1:**
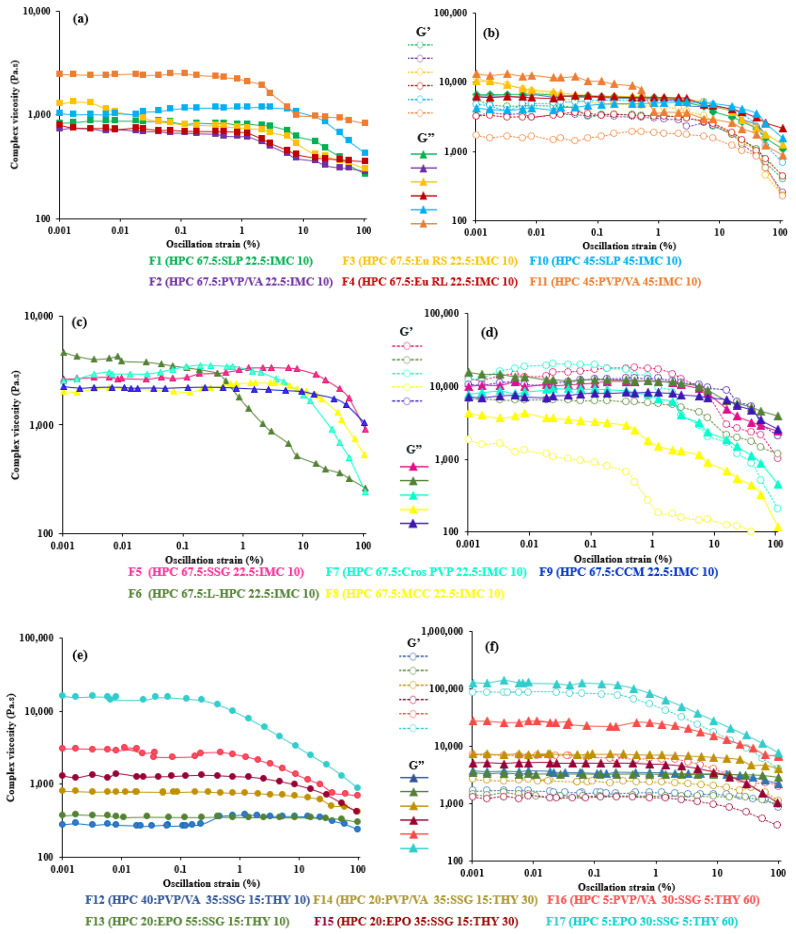
Effect of strain on complex viscosity (**left**) and storage (G’) and loss (G”) moduli as function strain (**right**) of (**a**,**b**) 67.5–45% HPC:polymer mixtures, (**c**,**d**) 67.5% HPC:disintegrant mixtures and (**e**,**f**) 40–5% HPC: polymer mixtures at 200 °C.

**Figure 2:**
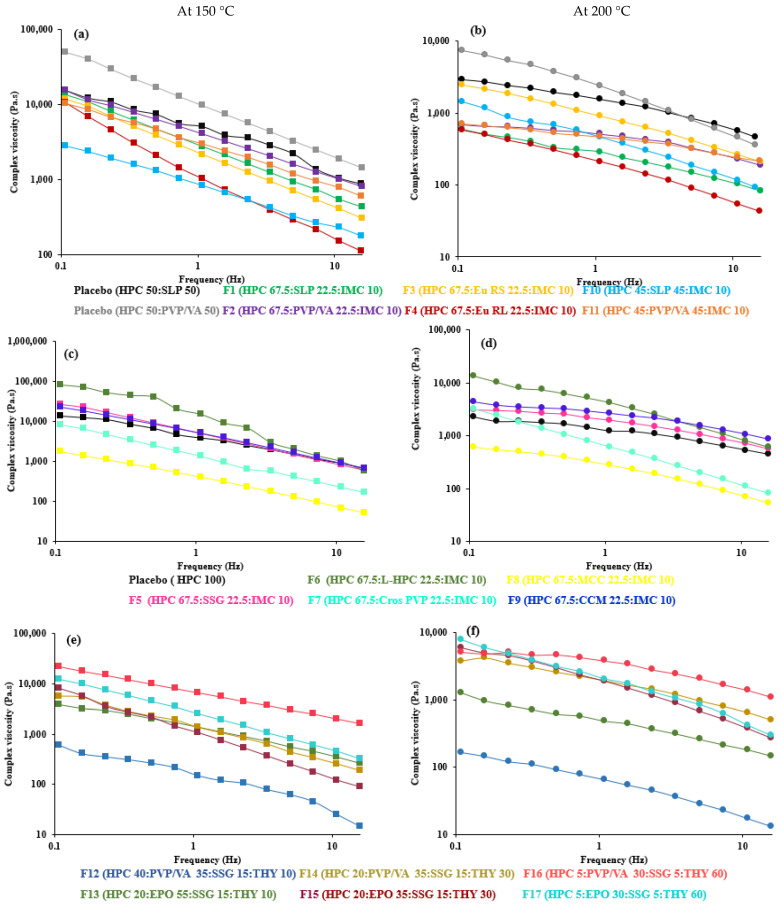
Effect of angular frequency (0.1 to 15 Hz) on complex viscosity of 67.5–45% HPC:polymer mixtures (**a**,**b**), 67.5% HPC:disintegrant mixtures (**c**,**d**) and 40–5% HPC:polymer mixtures (**e**,**f**) at 150 °C (**left**) and 200 °C (**right**).

To investigate the properties of the inherent material in a rheological test, it is crucial that the measurements are performed in the linear viscoelastic range, i.e., the deformation is maintained at a small level [62]. Thus, all tests are carried out within the LVER to evaluate the influence of the parameters other than stress or strain (such as frequency or temperature) on the shear moduli of the materials. As seen from Figure 1 and Figure 2, the oscillatory shear property (e.g., complex viscosity (η*) of most of the polymer–polymer blends filaments) is not dependent on the strain amplitude, γ°, and showed a wide LVER range from 0.001 to 40% strain, whereas that of the polymer–disintegrant blend filaments deviated from LVER at a high deformation indicated strain thinning (0.001–6%). This may be due to the behavior of the concentrated filler systems [64]. It can also be clearly seen in the formulations of F14–F17, which had high filler contents consecutively increasing the solid particle content significantly, which narrowed down the LVER that can be observed in the mixtures with a 60% theophylline content (F16 and F17: 0.001–0.5%), suggesting that they form a concentrated solid suspension under the recent measurement temperature [18]. This is consistent with general trends reported for nanocomposites and composite materials [47,65]. It may be concluded that the samples used in our study are linear viscoelastic up to 10% strain, except highly solid particle filled systems.

In all formulations, the (elastic) storage modulus, G’, and (viscous) loss modulus, G”, became a function of the strain amplitude, usually decreasing with increasing strain amplitude when the LVER is exceeded. These structural changes lead to the nonlinearity. All polymer–polymer filaments exhibited a predominantly viscous character with G’ < G”. However, in some formulations, such as F5, F7, F9 and F10, the elastic modulus (G’) was larger than the viscous one (G”) from the beginning of the test. The introduction of disintegrants in HPC was found to monotonically increase the viscoelastic properties compared to that of polymeric filaments, as expected for filled polymeric systems [47,65]. Likewise, a similar trend was observed in high theophylline-loaded formulations (undissolved theophylline).

### 3.2. Viscosity

The viscosity of the polymer melt is controlled by the presence of additive(s), as well as the temperature and shear rate applied in the melt extrusion, and it is, therefore, important to measure the effect of the temperature and angular frequency on the flow behavior and deformation of the molten mass. However, using oscillatory rheology is not able to specifically simulate the shear rate and pressure generated by the rotating screw/filament push during melt extrusion, because the shear rate produced by the oscillatory rheometer is often much lower. Nevertheless, it is possible to assess a preliminary evaluation of the effect of the shear rate on the viscosity by measuring the complex viscosity as a function of the angular frequency at a predetermined temperature and oscillation strain.

For successful HME, the melt viscosity of the material should be in a suitable range to rapidly dissolve the drug in the polymer melt during the extrusion process and to obtain the extruded filament that remains semi-solid after being out of the die. The relatively low viscosity enables the polymer to pass through the extruder without producing too high a torque. Nonetheless, the viscosity of the polymer melt should not be too low to form liquid extrudates or to start dripping from the printing nozzle, while housing the filament and waiting for the nozzle to be heated up to the designated temperature [20]. In previous studies, the complex viscosity of the melt was reported in the range 1000 to 10,000 Pa·s at the angular frequency of 0.1 rad/s for optimal hot melt extrusion [60,61,66,67]. In contrast, this study revealed a larger window of processing viscosities for the filaments used for successful printing. Although material-extrusion printing is in a similar process category to HME, the mode of shearing is different between these two processes. The shear stress is generated by the screw configuration (in the HME) and the driving gear (in the material-extrusion printing). Thus, a viscosity appropriate for HME may not be appropriate for material-extrusion printing.

The complex viscosity profiles as a function of angular frequency at HME and material-extrusion processing temperatures are shown in Figure 2(left). Increasing the angular frequency or temperature resulted in a decrease in viscosity for all the filaments.

At the temperature of 150 °C, where F1–F11 were successfully extruded for material extrusion printing, the complex viscosity of the blends at a low frequency (0.1 rad/s) was between 1721 and 188,783 Pa·s (Table 2), which may be considered to be acceptable for melt extrusion. This range is broader than previously reported [60], in particular, the upper limit. The greater complex viscosity could be attributed to the impeded polymer mobility as a consequence of the increased drug–filler–matrix interactions [68]. The lower viscosity of 45% HPC (F10 and F11) compared to 67.5% HPC (F1 and F2) may be linked to the reduced Tg of the blends (Tg of HPC [55], SLP [69] and PVP/VA [52] = 120, 70 and 104 °C, respectively) and the decreased polymeric chain interaction [47]. The addition of 22.5% L-HPC (F6) to HPC blends led to the highest viscosity among disintegrants, possibly owing to the presence of a solid filler [70,71] and the high Tg of the L-HPC (126 °C) [72]. However, with different drugs, drug loads and polymer composites, different extrusion conditions were required. An increase in drug content from 10 (F1–F13) to 60% (F14–F17) and a decrease in HPC from 67.5 to 5% needed a greater extrusion temperature, although their viscosity at 150 °C was in the aforementioned range. The temperature of 160 °C lowered the melt viscosity of F14–F17 to the range of 17,776 to 168,311 Pa·s (Table 2). Meanwhile, the screw speed used for F14–F17 was increased to adjust the filament diameter. The increased screw speed could increase the shear rate inside the extrusion barrel and, thus, further lower the melt viscosity.

From these findings, the viscosity range at the highest angular frequency could be a better representative to simulate the shear action during extrusion [73] than the range at 0.1 rad/s, as previously reported. The viscosity of the printable filaments at 100 rad/s was lowered to 42–1134 Pa·s for optimal HME (Table 2). At the temperature of 200 °C, where F1–F17 can be printed well and achieve sufficient material flow from the FDM 3D printer hot nozzle for successful 3D printing [32], the complex viscosity of all formulations reduced sharply and were in the range 295–31,445 Pa·s (at 0.1 rad/s) or 13–1099 (at 100 rad/s), which agrees with previous studies suggesting that printable polymeric systems possess complex viscosity less than 8000 Pa·s [32], less than 1200 Pa·s [51] or are in the order of 10^2^ Pa·s [34,39] at 100 rad/s. On the other hand, Elbadawi et al. and Boetker et al. found that filaments with a viscosity in the orders of 10^3^ and 10^4^ Pa·s were suitable for FDM printing [20,50]. Until now, the ideal viscosity for material extrusion printing has only been investigated in a limited way [73], and these data could be one piece of evidence.

Considering the same polymer composites (F14–F17), a greater viscosity was found with the higher drug loading concentrations at all temperatures. This could be due to the limited plasticizing effect of THY and the remaining undissolved THY in the ternary mixtures [74] with 30% and 60% drug loads, which reduced the interaction between the polymer and drug [70] when the drug solubility limit was passed [14]. It was proposed that, when the drug substance is not dissolved in the polymer and remains as a crystalline substance in the polymer matrix, it acts as a solid filler and raises the viscosity of the drug–polymer mixture [14,18,75,76]. It is possible that the plasticizing effect of the dissolved theophylline could not compensate for the thickening effect of the non-dissolved theophylline particles, and the undissolved THY did not melt at the extrusion temperatures to increase the viscosity since the melting point of THY is ca. 273 °C [66]. Some particles of the solid filler possibly form networks whose breaking down needs a specific amount of stress, which causes a considerable increase in the viscosity at a low deformation rate [47].

### 3.3. Shear Thinning

An ideal filament for material extrusion printing has a transition from Newtonian to shear thinning behavior and exhibits a significant shear thinning effect that results in optimal flowability out of the nozzle [5,77]. Shear thinning behavior influences not only the ability to be pushed through a narrow nozzle at a given temperature, but also the capability to regain structure after the shear discontinuance (post-deposition) [77]. Materials with high shear thinning, or *n*-value, have a lower tendency to back-flow within the nozzle [5,78]. Moreover, materials in a hot-end nozzle undergo moderately turbulent flow, which could hold a high level of back-mixing, and it then let the material experience a long residence time inside the nozzle, leading to a high thermal load on the material [79]. As such, materials with higher *n*-values potentially show less back-flow and back-mixing, which therefore reduces the thermal load to the API [33].

All the formulations exhibited a shear thinning effect over the entire frequency (Figure 2, right), indicating that the viscosity of the polymeric mixtures was influenced by the shear rate [66], as the shear load causes molecular disentanglement of the long polymer chains [80]. Meanwhile, in HPC-disintegrant blends, shear thinning was possibly attributed to the breakdown or disintegration of solid agglomerates [81]. These circumstances resulted in less resistance to flow at a higher frequency, which mimics the shear action in HME and FDM. In addition, the reduction in viscosity upon increasing angular frequency can be explained by comparing the slopes of the graph (Table 2). It was evident that the degree of viscosity reduction was different among the various polymer composites [67]. The lower slopes of viscosity vs. frequency from −0.1 to −0.4 gave rise to a high torque during processing, while the higher slopes allowed a larger temperature window for the processing of the polymer [67]. Herein, the slopes of all formulations ranged from −0.5 to −1 at 150 and 160 °C; however, the temperature window for HME was also limited by the diameter of the extruded filaments. Increasing the temperature resulted in a decrease in the slopes for all the filaments. At 200 °C for FDM printing, the slopes of all formulations ranged from −0.3 to −0.7, which is a broader range than in Ilyes’ work (−0.4 to −0.6) [51]. Greater shear thinning behavior or a higher slope value could be caused by the predominant interactions between the additive and polymer, and the breakage of the HPC polymer networks at a high frequency [68,82]. The addition of CrosPVP showed the greatest shear-thinning effect among the disintegrants upon printing (200 °C). Moreover, with 30 and 60% drug loads, PVP/VA mixtures (F14 and F16) showed less shear-thinning effects (less slope) than the EPO mixtures (F15 and F17), reflecting that EPO composites are more process-friendly.

### 3.4. Viscoelastic Properties of HME-and FDM-Printable Mixtures

Most pharmaceutical polymers have viscoelastic characteristics in nature, which implies that they have both solid-like and liquid-like features at various temperatures [66]. The storage modulus (G’) and loss modulus (G”) are measures of elastic and viscous responses in a polymer, respectively [27]. The storage modulus is used to support insight into the polymer elasticity [27] and entanglement [83], while the loss modulus is the replication of the energy dissipated, as obtained from the molecular friction [27]. These characters are crucial for both the printing quality and processability of HME and FDM. The printing quality of samples with the FDM technique corresponds to the viscoelastic properties outside the printing machine [84]. If G” is higher than G’ at all frequencies measured, the polymer solution behaves similar to a viscous material, and the printed material would generally maintain its shape [85]. Crossover (G’ = G”) is the point at which the storage modulus and loss modulus meet, and it defines the transition from a solid- to a liquid-dominated behavior. During printing, this relates to the time that the polymer is extruded through the nozzle. Therefore, the polymer must not have so large a storage modulus (solid-like behavior) that it cannot be extruded and becomes clogged, but the loss modulus (liquid-like behavior) should not be too low to facilitate the material to drain freely from the nozzle or fail to hold its shape [73]. The differences in the viscoelastic properties between the polymeric blends were observed, since the ratios of the polymers varied, but also with respect to their molecular weight (MW) and chain length [86]. Moreover, each excipient plays an essential part in the rheological behavior, including the API itself [51], as exemplified by the significant complex viscosity difference between the HPC in combination with different polymers and drug concentration.

The printable filaments must possess suitable rheological properties and mechanical strength to ensure optimal processability in both HME and FDM printing [87,88]. These two require viscosity matching to form layer uniformity in the barrel and die. However, the filament viscosity for FDM has to be much lower than that for hot melt extrusion for effective printing [61]. Since the filaments in FDM are influenced by the temperature-dependent phase transformation, with the additional piston-like effect of the drive-gear-driven filament column, as well as through a much smaller tip. The flow behaviors of the filaments are normally illustrated as the viscoelastic properties under stress and heat. The higher the storage modulus, the more elastic the sample, and the more difficult it will be to flow from the nozzle of a printer [89]. This can lead to the nozzle clogging before printing, and after printing, it will be the most rigid structure. Conversely, if this modulus is too low, the polymer will space out and may not withstand oscillation.

**Figure 3:**
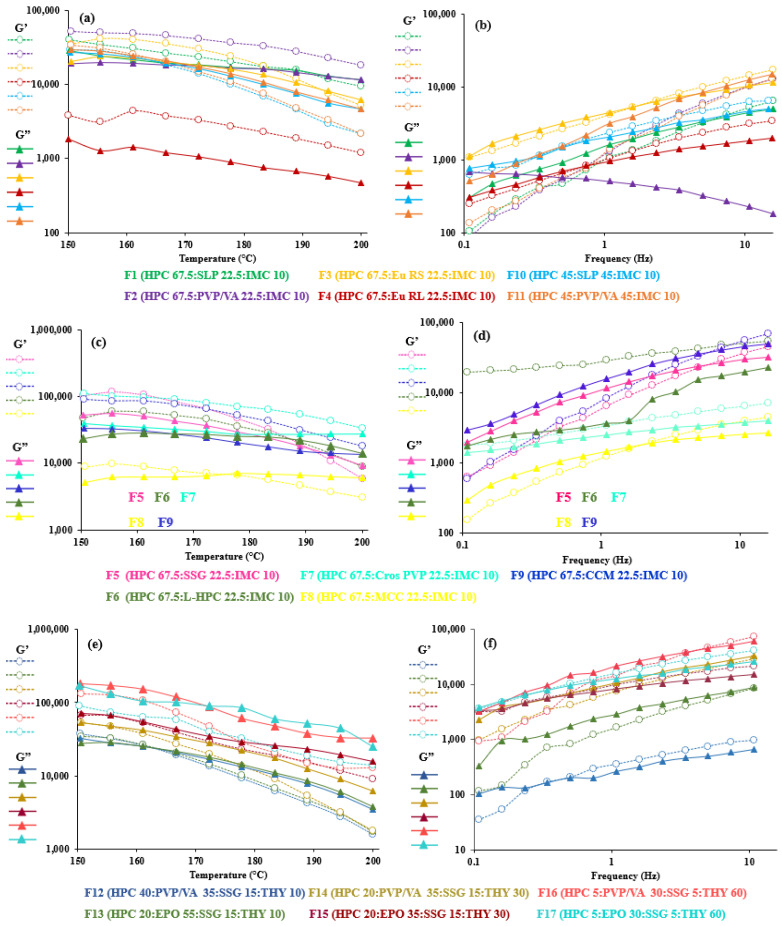
Viscoelastic properties (storage modulus (G’) and loss modulus (G’’)) of 67.5–45% HPC: polymer mixtures (**a**,**b**), 67.5% HPC:disintegrant mixtures (**c**,**d**) and 40–5% HPC:polymer mixtures (**e**,**f**) upon increasing temperatures at 1 Hz (**left**) and at 200 °C (**right**).

At the extrusion temperature (Figure 3, left), all the printable formulations of IMC (F1–F11) and of 10% THY (F12 and F13) exhibited dominantly elastic behavior (G’ > G”), while 30 and 60% THY (F14–F17) showed a more viscous character (G” > G’). It showed the viscoelasticity difference in the non-melt drug acting as a filler from the melt-blending formula. Since the THY did not melt at the extrusion temperatures (melting point ca. 273 °C), it could act as a filler and reduce the interaction between the polymer and drug, resulting in a lower viscosity [70]. At high filler content, some particles seemed to form networks whose breaking down needs a specific amount of stress, which causes a considerable increase in viscosity at a low deformation rate [47]. Van Renterghem et al. previously proposed that, when the drug substance is not dissolved in the polymer and remains as a crystalline substance in the polymer matrix, it acts as a solid filler and raises the viscosity of the drug–polymer mixture [75]. Similar observations were also reported previously by Suwardie et al. [14] and Yang et al. [76]. F10 and F11 showed more viscous characters (G” > G’) after the crossover temperature (161 and 167 °C, respectively). This suggested that the increase in SLP and PVA/VA altered the interaction and entanglement of the filament when compared to F1 and F12, with no crossover.

Nonetheless, MCC (F8) could be a good additive for HME and FDM. It has mostly been used in a formulation undergoing extrusion spheronization, as it provides good binding property, low friability and smooth surface properties [90]. This resulted from its filamentous structure with a large surface and high internal porosity [91]. MCC could also generate capillaries with other compacted compounds leading to wicking disintegration [92]. In other word, the intra-interaction of MCC is much lower than with other disintegrants. Our results showed clearly that the viscoelastic property of F8 was the lowest value, and its loss modulus, G”, was stable between 150 and 200 °C. In contrast to other fillers, there were interactions between the polymers and filler at some level; thus, their viscosities and viscoelasticity were higher. It is likely that the introduction of disintegrants in HPC polymers was found to monotonically increase the viscoelastic behavior compared to polymeric blends, and this is in agreement with general trends for filled polymeric systems [47,65].

To demonstrate the relationship between the viscosity property and all FDM-printable filaments, the oscillation frequency sweep test at 200 °C (Figure 3, right) was constructed. We would expect that G” should be greater than G’ to facilitate the polymer blends flowing through the nozzle easily and be simulated at a high frequency. The elastic-dominated trait (G” < G’) at a low frequency sweep could lead to a rigid structure after printing [85]. All 17 polymer composites displayed a liquid-like behavior at a low frequency, except for the F6 and F7 blends, but at a high frequency (100 rad/s), all systems (except F11) exhibited a marked elasticity or more rigid-like structure after the crossover moduli. The phenomena may be due to the crystalline network in the drug or solid filler, which may obstruct the flow through the nozzle and produce clogging [30]; however, the printing process went smoothly in practice. This contradiction was also shown in the mixture of polyethylene oxide, PEG and THY [32]. Furthermore, an increase in the elastic modulus (G’) at a low frequency could be presumed with the disintegrant agglomeration [93], effectively acting as an inorganic filler. This is consistent with previous studies, which have reported that increasing the filler content caused an increase in G’ at low ω [94,95]. Thus, it could be said that the initial increase in elasticity was not enough to enhance the viscosity of the formulation at a low frequency [20].

**Figure 4:**
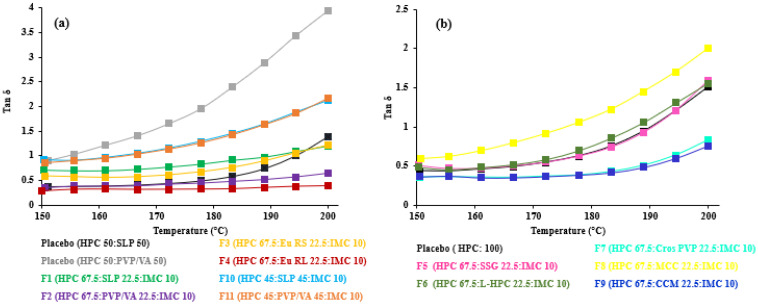

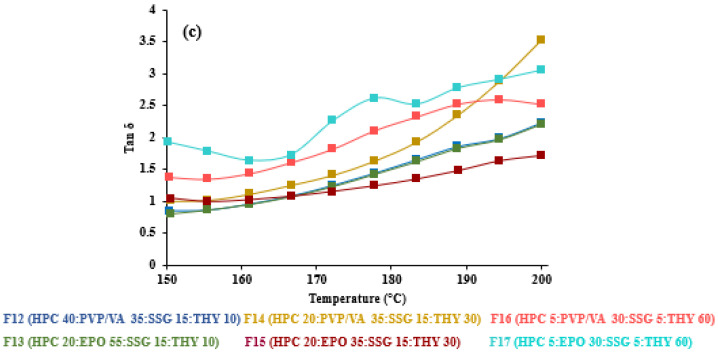
Tan delta vs. temperatures of (**a**) 67.5–45% HPC:polymer mixtures, (**b**) 67.5% HPC: disintegrant mixtures and (**c**) 40–5% HPC: polymer mixtures.

As seen in F12–F17, the drug load and polymer type affected the crossover frequency. At 30 and 60% THY concentrations, PVP/VA-based formulations (F14 and F16) had higher crossover frequencies than the EPO-based formulations (F15 and F17, Table 3). It is not always true that the crossover frequency decreased with an increase in drug loading due to the drug’s solubility in the polymer [14]. On the other hand, tan δ, the ratio of loss and storage moduli, showed noticeable transition of viscoelasticity, as shown in Figure 4. A value above one indicates that the sample is predominantly viscous, and below one, it is predominantly elastic [20]. Generally, the results revealed that the tan δ values decreased upon decreasing temperature and approached one. The formulations with low amount (less than 40%) of HPC (Figure 4c) led to the dominant viscous over 150–200 °C. In other words, high drug loading (60% THY, F16 and F17) in filaments retarded the composites to solidify, resulting in tan δ values of 1.4 and 2.0 at 150 °C, which are higher than those of low drug loading (30–10%, F12–F15) and placebo (Figure 4a,b).

When considering our oscillation temperature sweep, the higher temperature makes the filament more liquid-like, and it should be printable when having a G” more than G’. It could be a more suitable tool to predict the behavior of HME-FDM printable filament for true-behavior filler dispersion type, as seen in the results of F14–F17. In contrast to HME, where the extrusion process should be based on the tan δ value of 1, the preferable temperature for printing should have the tan δ value above one which may facilitate the flow of the polymer composite through the small printing nozzle. Moreover, the oscillation frequency sweep test could not determine which formula is printable for FDM. This proposed study provides an alternative to using rheological assessment to understand the FDM printing process of different formulations, even though no clear relationship was observed between the rheological properties and the printing behavior [33].

Future work is needed to study a more robust rheological dataset and its relationship between the material physical properties. From the polymer perspective, exploring the viscoelastic behaviors of polymer composites may lead to the insightful understanding on key material properties to foresee new composite systems for material-extrusion printing in the future. Moreover, viscosity is also related to the surface tension, due to the cohesion of the molecules in the fluid, and the surface tension is a property of the liquid, such that their surfaces behave similar to a thin film because of the inward pulling force exerted on the surface of the fluid. The increase in viscosity, due to the cohesion of the molecules in the fluid, would also increase the surface tension. Ding et al. proposed that the viscosity of polymer composites and the interfacial tension decreases with an increase in the temperature [96].

## 4. Conclusions

Material-extrusion printable filaments are required to flow at a high temperature inside the heated nozzle during printing. A rheometer was used to determine the complex viscosity and viscoelastic properties of the 17 blends for successfully printed immediate-and-controlled-release tablets. A decrease in the complex viscosity was observed for all polymer composites with an increase in temperature. A drop in viscosity by one order of magnitude was shown when increasing 50 °C temperature from 150 to 200 °C over the angular frequency. The influence of the shear rate on the viscosity can be seen from the slopes of the graphs. All formulations showed shear thinning behavior with a broad slope of complex viscosity from −0.28 to −0.74. A higher temperature showed less of an impact than the shear rate on the viscosity. However, the results demonstrated that the windows of processing viscosity were between 10 and 1000 Pa·s at a high frequency. No clear difference in viscosity and shear thinning character was seen between immediate and controlled release systems. Solid fillers, such as high drug or disintegrant content, could modify the viscosity, shear thinning and viscoelastic profiles, thus obtaining characteristic and intrinsic values for each formulation. HPC can be used in various ratios to obtain printable filaments, while MCC can be an alternative additive to lower the storage modulus and loss modulus among the disintegrants. Interestingly, high drug loading with 60% THY in filaments retarded the composites to solidify, resulting in tan δ values of 1.4 and 2.0. These new findings could be applied for developing other FDM-printed drug products in the future.

## Figures and Tables

**Table 1:** Optimized filament formulations and extrusion conditions.

Code	HPC (%*w*/*w*)	Polymer (%*w*/*w*)	Disintegrant (%*w*/*w*)	IMC (%*w*/*w*)	THY (%*w*/*w*)	Extrusion Temperature (°C)	Screw Speed (rpm)
F1	67.5	SLP, 22.5		10		150	35
F2	67.5	PVP/VA, 22.5		10		150	35
F3	67.5	Eu RS, 22.5		10		150	35
F4	67.5	Eu RL, 22.5		10		150	35
F5	67.5		SSG, 22.5	10		150	35
F6	67.5		L-HPC, 22.5	10		150	35
F7	67.5		CrosPVP, 22.5	10		150	35
F8	67.5		MCC, 22.5	10		150	35
F9	67.5		CCM, 22.5	10		150	35
F10	45	SLP, 45		10		150	35
F11	45	PVP/VA, 45		10		150	35
F12	40	PVP/VA, 35	SSG, 15		10	135	45
F13	20	EPO, 55	SSG, 15		10	135	35
F14	20	PVP/VA, 35	SSG, 15		30	160	45
F15	20	EPO, 35	SSG, 15		30	160	45
F16	5	PVP/VA, 30	SSG, 5		60	160	45
F17	5	EPO, 30	SSG, 5		60	160	45

**Table 2:** Comparison of complex viscosity and slopes of complex viscosity vs. oscillation frequency at different temperatures for 17 formulations.

Temperature	150 °C			160 °C			200 °C		
Code	Complex Viscosity (Pa·s) at 0.1 rad/s	Complex Viscosity (Pa·s) at 100 rad/s	Slope of Complex Viscosity	Complex Viscosity (Pa·s) at 0.1 rad/s	Complex Viscosity (Pa·s) at 100 rad/s	Slope of Complex Viscosity	Complex Viscosity (Pa·s) at 0.1 rad/s	Complex Viscosity (Pa·s) at 100 rad/s	Slope of Complex Viscosity
**F1**	31,346	429	−0.703	17,897	372	−0.636	968	82	−0.395
**F2**	33,669	799	−0.579	18,781	617	−0.546	856	185	−0.299
**F3**	32,346	304	−0.745	21,881	308	−0.693	3984	207	−0.511
**F4**	38,347	110	−0.906	19,636	190	−0.818	984	42	−0.564
**F5**	58,240	595	−0.786	47,888	556	−0.745	9334	555	−0.363
**F6**	188,783	567	−0.992	168,311	1457	−0.767	31,445	618	−0.555
**F7**	20,773	163	−0.799	19,870	138	−0.802	7380	82	−0.736
**F8**	5855	42	−0.814	5445	35	−0.721	1678	52	−0.511
**F9**	49,644	667	−0.731	45,230	594	−0.744	6684	855	−0.320
**F10**	4899	177	−0.572	3286	120	−0.580	2768	92	−0.582
**F11**	18,653	596	−0.567	18,334	744	−1.196	1200	218	−0.517
**F12**	1721	14	−0.693	1043	15	−0.680	295	13	−0.490
**F13**	5768	263	−0.518	4309	184	−0.524	3138	146	−0.424
**F14**	8923	186	−0.677	7089	394	−0.447	6522	498	−0.378
**F15**	23,865	89	−0.909	17,776	118	−0.708	9334	274	−0.629
**F16**	47,227	1630	−0.521	30,560	1134	−0.556	7522	1099	−0.284
**F17**	27,773	323	−0.718	22,887	465	−0.603	19,560	292	−0.636

**Table 3:** Crossover temperature and crossover frequency for all formulations.

Code	Crossover Temperature (°C) at Fixed Frequency (1 Hz)	Crossover Frequency (Hz) at 200 °C
F1	NC	5.09
F2	NC	0.50
F3	194	2.34
F4	NC	0.74
F5	190	5.03
F6	189	NC
F7	200	NC
F8	178	1.59
F9	NC	5.03
F10	161	0.50
F11	167	NC
F12	161	0.23
F13	161	7.38
F14	150	10.8
F15	150	0.34
F16	NC	3.43
F17	NC	0.23

NC—no crossover.

## References

[1] Goyanes A., Buanz A.B.M., Basit A.W., Gaisford S. (2014). Fused-filament 3D printing (3DP) for fabrication of tablets. Int. J. Pharm..

[2] Khaled S.A., Burley J.C., Alexander M.R., Roberts C.J. (2014). Desktop 3D printing of controlled release pharmaceutical bilayer tablets. Int. J. Pharm..

[3] Murugan S., Vinodh S. (2020). Parametric optimization of fused deposition modelling process using Grey based Taguchi and TOPSIS methods for an automotive component. Rapid Prototyp. J..

[4] Lalegani M., Mohd Ariffin M.K.A., Hatami S. (2021). An overview of fused deposition modelling (FDM): Research, development and process optimisation. Rapid Prototyp. J..

[5] Gilmer E.L., Miller D., Chatham C.A., Zawaski C., Fallon J.J., Pekkanen A., Long T.E., Williams C.B., Bortner M.J. (2018). Model analysis of feedstock behavior in fused filament fabrication: Enabling rapid materials screening. Polymer.

[6] Awad A., Gaisford S., Basit A.W., Basit A.W., Gaisford S. (2018). Fused Deposition Modelling: Advances in Engineering and Medicine. 3D Printing of Pharmaceuticals.

[7] Khorasani M., Ghasemi A., Rolfe B., Gibson I.J.R.P.J. (2021). Additive manufacturing a powerful tool for the aerospace industry. Rapid Prototyp. J..

[8] Ranjan N., Singh R., Ahuja I. (2020). Mechanical, Rheological and Thermal Investigations of Biocompatible Feedstock Filament Comprising of PVC, PP and HAp. Proc. Natl. Acad. Sci. India Sect. A Phys. Sci..

[9] Ching-hao L., Mohammad Padzil F.N., Lee S.H., Mohamed A.Z., Luqman Chuah A. (2021). Potential for Natural Fiber Reinforcement in PLA Polymer Filaments for Fused Deposition Modeling (FDM) Additive Manufacturing: A Review. Polymers.

[10] Zidan A., Alayoubi A., Coburn J., Asfari S., Ghammraoui B., Cruz C.N., Ashraf M. (2019). Extrudability analysis of drug loaded pastes for 3D printing of modified release tablets. Int. J. Pharm..

[11] Kempin W., Domsta V., Grathoff G., Brecht I., Semmling B., Tillmann S., Weitschies W., Seidlitz A. (2018). Immediate Release 3D-Printed Tablets Produced Via Fused Deposition Modeling of a Thermo-Sensitive Drug. Pharm. Res..

[12] Forster A., Hempenstall J., Tucker I., Rades T. (2001). Selection of excipients for melt extrusion with two poorly water-soluble drugs by solubility parameter calculation and thermal analysis. Int. J. Pharm..

[13] Liu H., Wang P., Zhang X., Shen F., Gogos C.G. (2010). Effects of extrusion process parameters on the dissolution behavior of indomethacin in Eudragit^®^ E PO solid dispersions. Int. J. Pharm..

[14] Suwardie H., Wang P., Todd D.B., Panchal V., Yang M., Gogos C.G. (2011). Rheological study of the mixture of acetaminophen and polyethylene oxide for hot-melt extrusion application. Eur. J. Pharm. Biopharm..

[15] Kalivoda A., Fischbach M., Kleinebudde P. (2012). Application of mixtures of polymeric carriers for dissolution enhancement of oxeglitazar using hot-melt extrusion. Int. J. Pharm..

[16] Sathigari S.K., Radhakrishnan V.K., Davis V.A., Parsons D.L., Babu R.J. (2012). Amorphous-State Characterization of Efavirenz—polymer Hot-Melt Extrusion Systems for Dissolution Enhancement. J. Pharm. Sci..

[17] Tadros T.F.J.L. (1990). Use of viscoelastic measurements in studying interactions in concentrated dispersions. Langmuir.

[18] Aho J., Edinger M., Botker J., Baldursdottir S., Rantanen J. (2016). Oscillatory Shear Rheology in Examining the Drug-Polymer Interactions Relevant in Hot Melt Extrusion. J. Pharm. Sci..

[19] De Brabander C., Van Den Mooter G., Vervaet C., Remon J.P. (2002). Characterization of ibuprofen as a nontraditional plasticizer of ethyl cellulose. J. Pharm. Sci..

[20] Elbadawi M. (2019). Rheological and Mechanical Investigation into the Effect of Different Molecular Weight Poly(ethylene glycol)s on Polycaprolactone-Ciprofloxacin Filaments. ACS Omega.

[21] Turner B., Gold S. (2015). A review of melt extrusion additive manufacturing processes: II. Materials, dimensional accuracy, and surface roughness. Rapid Prototyp. J..

[22] Greeff P., Schilling M. (2016). Closed Loop Control of Slippage during Filament Transport in Molten Material Extrusion. Addit. Manuf..

[23] Bellini A., Güçeri S.u., Bertoldi M. (2004). Liquefier Dynamics in Fused Deposition. J. Manuf. Sci. Eng..

[24] Zhang J., Feng X., Patil H., Tiwari R.V., Repka M.A. (2017). Coupling 3D Printing with Hot-Melt Extrusion to Produce Controlled-Release Tablets. Int. J. Pharm..

[25] Than Y.M., Titapiwatanakun V. (2021). Tailoring immediate release FDM 3D printed tablets using a quality by design (QbD) approach. Int. J. Pharm..

[26] Than Y.M., Titapiwatanakun V. (2021). Statistical design of experiment-based formulation development and optimization of 3D printed oral controlled release drug delivery with multi target product profile. J. Pharm. Investig..

[27] Aho J., Boetker J.P., Baldursdottir S., Rantanen J. (2015). Rheology as a Tool for Evaluation of Melt Processability of Innovative Dosage Forms. Int. J. Pharm..

[28] He P., Yu W., Zhou C. (2019). Agglomeration of Crystals during Crystallization of Semicrystalline Polymers: A Suspension-Based Rheological Study. Macromolecules.

[29] Defeng w., Wu L., Wu L., Zhang M. (2006). Rheology and thermal stability of polylactide/clay nanocomposites. Polym. Degrad. Stab..

[30] Calafel I., Aguirresarobe R.H., Peñas M.I., Santamaria A., Tierno M., Conde J.I., Pascual B. (2020). Searching for Rheological Conditions for FFF 3D Printing with PVC Based Flexible Compounds. Materials.

[31] Elbadawi M. (2018). Polymeric Additive Manufacturing: The Necessity and Utility of Rheology. Polymer Rheology.

[32] Isreb A., Baj K., Wojsz M., Isreb M., Peak M., Alhnan M.A. (2019). 3D printed oral theophylline doses with innovative ‘radiator-like’ design: Impact of polyethylene oxide (PEO) molecular weight. Int. J. Pharm..

[33] Henry S., Samaro A., Marchesini F.H., Shaqour B., Macedo J., Vanhoorne V., Vervaet C. (2021). Extrusion-based 3D printing of oral solid dosage forms: Material requirements and equipment dependencies. Int. J. Pharm..

[34] Elbadawi M., Gustaffson T., Gaisford S., Basit A.W. (2020). 3D printing tablets: Predicting printability and drug dissolution from rheological data. Int. J. Pharm..

[35] Killion J.A., Geever L.M., Devine D.M., Kennedy J.E., Higginbotham C.L. (2011). Mechanical properties and thermal behaviour of PEGDMA hydrogels for potential bone regeneration application. J. Mech. Behav. Biomed. Mater..

[36] Kim S.H., Yeon Y.K., Lee J.M., Chao J.R., Lee Y.J., Seo Y.B., Sultan M.T., Lee O.J., Lee J.S., Yoon S.-I. (2018). Precisely printable and biocompatible silk fibroin bioink for digital light processing 3D printing. Nat. Commun..

[37] Calvet D., Wong J., Giasson S. (2004). Rheological Monitoring of Polyacrylamide Gelation: Importance of Cross-Link Density and Temperature. Macromolecules.

[38] Erwin B., Cloitre M., Gauthier M., Vlassopoulos D. (2010). Dynamics and rheology of colloidal star polymers. Soft Matter.

[39] Coogan T., Kazmer D. (2019). In-line rheological monitoring of fused deposition modeling. J. Rheol..

[40] Ramos L. (2013). Principles of Polymer Processing.

[41] Fan B., Kazmer D.O., Nageri R. (2006). An Analytical Non-Newtonian and Nonisothermal Viscous Flow Simulation. Polym.-Plast. Technol. Eng..

[42] Ramanath H.S., Chua C., Leong K., Shah K.D. (2008). Melt flow behaviour of poly0µ-caprolactone in fused deposition modelling. J. Mater. Sci. Mater. Med..

[43] Cerda-Avila S., Medellín-Castillo H., Lim T. (2021). Analytical models to estimate the structural behaviour of fused deposition modelling components. Rapid Prototyp. J..

[44] Bakır A., Atik R., Özerinç S. (2021). Mechanical properties of thermoplastic parts produced by fused deposition modeling: A review. Rapid Prototyp. J..

[45] Vanaei H., Shirinbayan M., Vanaei S., Fitoussi J., Khelladi S., Tcharkhtchi A. (2020). Multi-scale Damage Analysis and Fatigue Behavior of PLA Manufactured By Fused Deposition Modeling (FDM). Rapid Prototyp. J..

[46] Fuenmayor E., Forde M., Healy A., Devine D., Lyons J., McConville C., Major I. (2018). Material Considerations for Fused-Filament Fabrication of Solid Dosage Forms. Pharmaceutics.

[47] Sadia M., Sośnicka A., Arafat B., Isreb A., Ahmed W., Kelarakis A., Alhnan M.A. (2016). Adaptation of pharmaceutical excipients to FDM 3D printing for the fabrication of patient-tailored immediate release tablets. Int. J. Pharm..

[48] Sadia M., Isreb A., Abbadi I., Isreb M., Aziz D., Selo A., Timmins P., Alhnan M.A. (2018). From ‘fixed dose combinations’ to ‘a dynamic dose combiner’: 3D printed bi-layer antihypertensive tablets. Eur. J. Pharm. Sci..

[49] Aho J., Genina N., Edinger M., Botker J.P., Baldursdottir S., Rantanen J. Drug-loaded poly (ε-caprolactone) for 3D printing of personalized medicine: A rheological study. Proceedings of the 25th Nordic Rheology Conference.

[50] Boetker J., Water J.J., Aho J., Arnfast L., Bohr A., Rantanen J. (2016). Modifying release characteristics from 3D printed drug-eluting products. Eur. J. Pharm. Sci..

[51] Ilyés K., Kovács N.K., Balogh A., Borbás E., Farkas B., Casian T., Marosi G., Tomuță I., Nagy Z.K. (2019). The applicability of pharmaceutical polymeric blends for the fused deposition modelling (FDM) 3D technique: Material considerations–printability–process modulation, with consecutive effects on in vitro release, stability and degradation. Eur. J. Pharm. Sci..

[52] Vo A.Q., Zhang J., Nyavanandi D., Bandari S., Repka M.A. (2020). Hot melt extrusion paired fused deposition modeling 3D printing to develop hydroxypropyl cellulose based floating tablets of cinnarizine. Carbohydr. Polym..

[53] Chai X., Chai H., Wang X., Yang J., Li J., Zhao Y., Cai W., Tao T., Xiang X.J.S.R. (2017). Fused Deposition Modeling (FDM) 3D Printed Tablets for Intragastric Floating Delivery of Domperidone. Sci. Rep..

[54] Gorkem Buyukgoz G., Soffer D., Defendre J., Pizzano G.M., Davé R.N. (2020). Exploring tablet design options for tailoring drug release and dose via fused deposition modeling (FDM) 3D printing. Int. J. Pharm..

[55] Öblom H., Zhang J., Pimparade M., Speer I., Preis M., Repka M., Sandler N. (2019). 3D-Printed Isoniazid Tablets for the Treatment and Prevention of Tuberculosis—Personalized Dosing and Drug Release. AAPS PharmSciTech.

[56] Tan D.K., Maniruzzaman M., Nokhodchi A. (2020). Development and Optimisation of Novel Polymeric Compositions for Sustained Release Theophylline Caplets (PrintCap) via FDM 3D Printing. Polymers.

[57] Korte C., Quodbach J. (2018). Formulation development and process analysis of drug-loaded filaments manufactured via hot-melt extrusion for 3D-printing of medicines. Pharm. Dev. Technol..

[58] Samaro A., Janssens P., Vanhoorne V., Van Renterghem J., Eeckhout M., Cardon L., De Beer T., Vervaet C. (2020). Screening of pharmaceutical polymers for extrusion-Based Additive Manufacturing of patient-tailored tablets. Int. J. Pharm..

[59] Arafat B., Wojsz M., Isreb A., Forbes R.T., Isreb M., Ahmed W., Arafat T., Alhnan M.A. (2018). Tablet fragmentation without a disintegrant: A novel design approach for accelerating disintegration and drug release from 3D printed cellulosic tablets. Eur. J. Pharm. Sci..

[60] Gupta S.S., Meena A.K., Parikh T., Serajuddin A.T. (2014). Investigation of thermal and viscoelastic properties of polymers relevant to hot melt extrusion, I: Polyvinylpyrrolidone and related polymers. J. Excip. Food Chem..

[61] Solanki N.G., Tahsin M., Shah A.V., Serajuddin A.T.M. (2018). Formulation of 3D Printed Tablet for Rapid Drug Release by Fused Deposition Modeling: Screening Polymers for Drug Release, Drug-Polymer Miscibility and Printability. J. Pharm. Sci..

[62] Amorim P.A., d’Ávila M.A., Anand R., Moldenaers P., Van Puyvelde P., Bloemen V. (2021). Insights on shear rheology of inks for extrusion-based 3D bioprinting. Bioprinting.

[63] Diab A., You Z. (2017). Small and large strain rheological characterizations of polymer- and crumb rubber-modified asphalt binders. Constr. Build. Mater..

[64] Mueller S., Llewellin E., Mader H., Mueller B., Mader A. (2009). The rheology of suspensions of solid particles. Proc. R. Soc. A Proc. R. Soc. A.

[65] Yurekli K., Krishnamoorti R., Tse M., McElrath K., Tsou A., Wang H. (2001). Structure and dynamics of carbon black-filled elastomers. J. Polym. Sci. Part B Polym. Phys..

[66] Gupta S.S., Parikh T., Meena A.K., Mahajan N., Vitez I., Serajuddin A.T.M. (2015). Effect of carbamazepine on viscoelastic properties and hot melt extrudability of Soluplus^®^. Int. J. Pharm..

[67] Gupta S.S., Solanki N., Serajuddin A.T.M. (2016). Investigation of Thermal and Viscoelastic Properties of Polymers Relevant to Hot Melt Extrusion, IV: Affinisol™ HPMC HME Polymers. AAPS PharmSciTech.

[68] Lee K., Brandt M., Shanks R., Daver F. (2020). Rheology and 3D Printability of Percolated Graphene-Polyamide-6 Composites. Polymers.

[69] Hardung H., Djuric D., Ali S. (2010). Combining HME & Solubilization: Soluplus^®^—The Solid Solution. Drug Deliv. Technol..

[70] Solanki N., Gupta S.S., Serajuddin A.T.M. (2018). Rheological analysis of itraconazole-polymer mixtures to determine optimal melt extrusion temperature for development of amorphous solid dispersion. Eur. J. Pharm. Sci..

[71] Kaully T., Siegmann A., Shacham D. (2007). Rheology of highly filled natural CaCO_3_ composites. II. Effects of solid loading and particle size distribution on rotational rheometry. Polym. Compos..

[72] Gómez-Carracedo A., Alvarez-Lorenzo C., Gómez-Amoza J.L., Concheiro A. (2003). Chemical structure and glass transition temperature of non-ionic cellulose ethers. J. Therm. Anal. Calorim..

[73] Azad M.A., Olawuni D., Kimbell G., Badruddoza A.Z., Hossain M.S., Sultana T. (2020). Polymers for Extrusion-Based 3D Printing of Pharmaceuticals: A Holistic Materials–Process Perspective. Pharmaceutics.

[74] Tidau M., Kwade A., Finke H.J. (2019). Influence of High, Disperse API Load on Properties along the Fused-Layer Modeling Process Chain of Solid Dosage Forms. Pharmaceutics.

[75] Van Renterghem J., Vervaet C., De Beer T. (2017). Rheological Characterization of Molten Polymer-Drug Dispersions as a Predictive Tool for Pharmaceutical Hot-Melt Extrusion Processability. Pharm. Res..

[76] Yang F., Su Y., Zhang J., DiNunzio J., Leone A., Huang C., Brown C.D. (2016). Rheology Guided Rational Selection of Processing Temperature To Prepare Copovidone–Nifedipine Amorphous Solid Dispersions via Hot Melt Extrusion (HME). Mol. Pharm..

[77] Nguyen N.A., Bowland C.C., Naskar A.K. (2018). A general method to improve 3D-printability and inter-layer adhesion in lignin-based composites. Appl. Mater. Today.

[78] Mackay M.E. (2018). The importance of rheological behavior in the additive manufacturing technique material extrusion. J. Rheol..

[79] Feuerbach T., Kock S., Thommes M. (2018). Characterisation of fused deposition modeling 3D printers for pharmaceutical and medical applications. Pharm. Dev. Technol..

[80] Vlachopoulos J., Strutt D., Wagner J.R. (2016). 6-Rheology of Molten Polymers. Multilayer Flexible Packaging.

[81] Behzadfar E., Abdolrasouli M.H., Sharif F., Nazockdast H. (2009). Effect of solid loading and aggregate size on the rheological behavior of PDMS/Calcium Carbonate suspensions. Braz. J. Chem. Eng..

[82] Pryamitsyn V., Ganesan V. (2006). Mechanisms of steady-shear rheology in polymer-nanoparticle composites. J. Rheol..

[83] Zhao H., Yan X., Zhao G., Guo Z. (2016). Microcellular Injection Molded Polylactic Acid/Poly (ε-Caprolactone) Blends with Supercritical CO_2_: Correlation between Rheological Properties and Their Foaming Behavior. Polym. Eng. Sci..

[84] Cicala G., Giordano D., Tosto C., Filippone G., Recca A., Blanco I. (2018). Polylactide (PLA) Filaments a Biobased Solution for Additive Manufacturing: Correlating Rheology and Thermomechanical Properties with Printing Quality. Materials.

[85] Hu Y., Wang J., Li X., Hu X., Zhou W., Dong X., Wang C., Yang Z., Binks B.P. (2019). Facile preparation of bioactive nanoparticle/poly(ε-caprolactone) hierarchical porous scaffolds via 3D printing of high internal phase Pickering emulsions. J. Colloid Interface Sci..

[86] Meena A., Parikh T., Gupta S.S., Serajuddin A. (2014). Investigation of thermal and viscoelastic properties of polymers relevant to hot melt extrusion—II: Cellulosic polymers. J. Excip. Food Chem..

[87] Konta A.A., García-Piña M., Serrano D.R. (2017). Personalised 3D Printed Medicines: Which Techniques and Polymers Are More Successful?. Bioengineering.

[88] Goyanes A., Det-Amornrat U., Wang J., Basit A.W., Gaisford S. (2016). 3D Scanning and 3D Printing as Innovative Technologies for Fabricating Personalized Topical Drug Delivery Systems. J. Control Release.

[89] Polamaplly P., Cheng Y., Shi X., Manikandan K., Kremer G.E., Qin H. (2019). 3D Printing and Characterization of Hydroxypropyl Methylcellulose and Methylcellulose for Biodegradable Support Structures. Procedia Manuf..

[90] Nguyen T.T.L., Anton N., Vandamme T.F., Andronescu E., Grumezescu A.M. (2017). Oral pellets loaded with nanoemulsions. Nanostructures for Oral Medicine.

[91] Shah R.D., Kabadi M., Pope D.G., Augsburger L.L. (1995). Physico-Mechanical Characterization of the Extrusion-Spheronization Process. Part II: Rheological Determinants for Successful Extrusion and Spheronization. Pharm. Res. Off. J. Am. Assoc. Pharm. Sci..

[92] Rojas J., Guisao S., Ruge V. (2012). Functional assessment of four types of disintegrants and their effect on the spironolactone release properties. AAPS PharmSciTech.

[93] Scoutaris N., Ross S., Douroumis D. (2018). 3D Printed “Starmix” Drug Loaded Dosage Forms for Paediatric Applications. Pharm. Res..

[94] Wu G., Zheng Q. (2004). Estimation of the Agglomeration Structure for Conductive Particles and Fiber-Filled High-Density Polyethylene through Dynamic Rheological Measurements. J. Polym. Sci. Part B: Polym. Phys..

[95] Moniruzzaman M., Winey K. (2006). Polymer Nanocomposites Containing Carbon Nanotubes. Macromolecules.

[96] Ding Y., Abeykoon C., Perera Y.S. (2022). The effects of extrusion parameters and blend composition on the mechanical, rheological and thermal properties of LDPE/PS/PMMA ternary polymer blends. Adv. Ind. Manuf. Eng..

